# Prevalence and associated factors for non-alcoholic fatty liver disease among adults in the South Asian Region: a meta-analysis

**DOI:** 10.1016/j.lansea.2023.100220

**Published:** 2023-05-24

**Authors:** Madunil Anuk Niriella, Dileepa Senajith Ediriweera, Madhuri Yasodha Withanage, Selani Darshika, Shamila Thivanshi De Silva, Hithanadura Janaka de Silva

**Affiliations:** a Faculty of Medicine, Department of Medicine, University of Kelaniya, Ragama, Sri Lanka; b Health Data Science Unit, Faculty of Medicine, University of Kelaniya, Ragama, Sri Lanka

**Keywords:** Meta-analysis, Prevalence, Pooled prevalence, Associations, NAFLD, Non-alcoholic fatty liver disease, South Asia

## Abstract

**Background:**

Non-alcoholic fatty liver disease (NAFLD) is the commonest chronic liver disease worldwide. We estimated the prevalence and predefined associated factors for NAFLD among South-Asian adults.

**Methods:**

We searched PubMed and included descriptive, epidemiological studies with satisfactory methodology, reporting the prevalence of NAFLD with ultrasound. Two authors screened and extracted data independently. Gender, urban/rural settings, general population and individuals with metabolic diseases (MetD) stratified the analysis. In addition, a random-effects meta-analysis of the prevalence and effect sizes of associations of NAFLD was performed.

**Findings:**

Twenty-two publications were included after the quality assurance process. The difference in the NAFLD prevalence between the general population and people with MetD was found to be statistically significant (Q = 15.8, DF = 1, P < 0.001). The pooled overall prevalence of NAFLD in the general population was 26.9% (95% CI: 18.9–35.8%) with high heterogeneity. The prevalence was similar among men and women (Q = 0.06, DF = 1, P = 0.806). The NAFLD prevalence in the rural communities was 22.6% (95% CI: 13.6–33.1%), and the prevalence in urban communities was 32.9% (95% CI: 22.8–43.8%) and the difference was not statistically significant (Q = 1.92, DF = 1, P = 0.166). The pooled overall prevalence of NAFLD in patients with MetD was 54.1% (95% CI: 44.1–63.9%) with high heterogeneity. The pooled overall prevalence of NAFLD in the non-obese population was 11.7% (95% CI: 7.0–17.3%). The pooled prevalence of non-obese NAFLD in the NAFLD population was 43.4% (95% CI: 28.1–59.4%). Meta-analysis of binary variables showed that NAFLD in the South Asian population was associated with diabetes mellitus, hypertension, dyslipidaemia, general obesity, central obesity and metabolic syndrome. Gender was not associated with NAFLD.

**Interpretation:**

The overall prevalence of NAFLD among adults in South Asia is high, especially in those with MetD, and a considerable proportion is non-obese. In the South Asian population, NAFLD was associated with diabetes mellitus, hypertension, dyslipidaemia, general obesity, central obesity, and metabolic syndrome.

**Funding:**

None.

**Research in context**

**Evidence before this study**

The prevalence of non-alcoholic fatty liver disease (NAFLD) in South Asian countries is high. There have been two previous systematic reviews on NAFLD from the South Asian region. These studies evaluated NAFLD’s prevalence, associations, and phenotype in South Asian countries, including Bangladesh, India, Nepal, Pakistan, and Sri Lanka. However, there has been no meta-analysis of epidemiological data on NAFLD from this region.

**Added value of this study**

The present study is the only meta-analysis on the prevalence of NAFLD in the South Asian region. The meta-analysis comprehensively assesses the available data over 17 years (2004–2021). The prevalence of NAFLD among adults in the South Asian region seems comparable to the global average. A considerable proportion of adults were found to be non-obese and NAFLD prevalence was notably higher among individuals with metabolic abnormalities. Diabetes mellitus, hypertension, dyslipidaemia, metabolic syndrome, and obesity (general and central) were associated with NAFLD in the South Asian region.

**Implications of all the available evidence**

The present study highlights the growing burden on NAFLD in the South Asian region, the most populous and densely populated geographical region in the world, with a population of 1.9 billion. Targeted health strategies should be implemented across the South-Asian region to address this growing public health problem of NAFLD and its long-term medical consequences.

## Introduction

Non-alcoholic fatty liver disease (NAFLD) is the commonest chronic liver disease worldwide.^1^ NAFLD is characterised by hepatic steatosis detected by either imaging or histology without secondary causes. It spans a spectrum of the disease from non-alcoholic fatty liver (NAFL) to non-alcoholic steatohepatitis (NASH) and ultimately to cirrhosis and its complications.^2^ Most people have a simple fatty liver with no or mild non-specific inflammation, without liver fibrosis. Conversely, NASH, the more severe form of the disease, has varying degrees of liver fibrosis, resulting in cirrhosis and its complications and comorbid cardiovascular disease.^3^

Recent studies report the global pooled prevalence of NAFLD at 25.2%, with wide geographical variation worldwide.^1^ The highest prevalence rates of up to 30%, primarily based on abdominal ultrasound, are from the Middle East and South American countries.^4^ A study by Ge and colleagues showed an association between NAFLD prevalence and overweight status and diabetes.^5^ Whilst a large part of the global increase in NAFLD is driven by obesity,^4^ patterns of increased prevalence do not always associate with areas of higher caloric consumption, suggesting that other factors may contribute to the progression of NAFLD.^6^^,^^7^

The prevalence of NAFLD in South Asian countries is high,^8^^,^^9^ which is presumably due to several factors such as socioeconomic growth, urbanisation, westernised diet, increasingly sedentary lifestyle and poor health awareness.^9^ Almost 20% of the world’s population resides in South Asia, which renders it the most densely populated region with many patients with NAFLD and metabolic syndrome.^9^

There have been two previous systematic reviews on NAFLD from the South Asian region. These studies evaluated NAFLD’s prevalence, associations, and phenotype in South Asian countries, including Bangladesh, India, Nepal, Pakistan, and Sri Lanka. However, there has yet to be a meta-analysis of epidemiological data on NAFLD from this region.

In the present paper, studies on the prevalence of NAFLD and its associations in South Asian countries up to May 2021 were searched and included in a meta-analysis. The aim was to estimate the overall prevalence and effect sizes of the associated pre-defined co-factors for NAFLD among adults in the South Asian region.

## Methods

### Search strategy

**Fig. 1:**
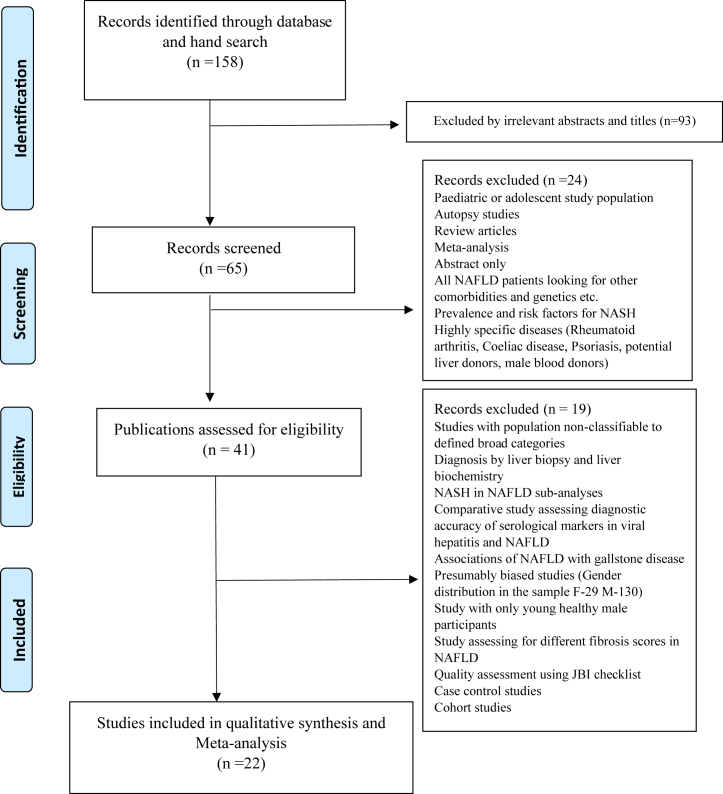
**PRISMA flowchart for selection of studies**. Abbreviation: NAFLD: non-alcoholic fatty liver disease.

A comprehensive survey on PubMed was carried out for articles published up to May 2021 with information on the prevalences and associated factors for NAFLD in South Asia. The search terms included “Prevalence of NAFLD (Non-Alcoholic Fatty Liver Disease)” AND “South Asia” AND individual South Asian countries (“Afghanistan”, “Bangladesh”, “Bhutan”, “India”, “Maldives”, “Nepal”, “Pakistan” and “Sri Lanka”). For reporting, we followed the preferred reporting items for systematic reviews and meta-analysis (PRISMA) guidelines for the conduct of this study (Fig. 1).

### Inclusion and exclusion criteria

The following inclusion criteria were used to select eligible articles published before May 2021: (1) descriptive, epidemiological studies reporting NAFLD, (2) studies conducted in the South Asian region (Afghanistan, Bangladesh, Bhutan, India, Maldives, Nepal, Pakistan, and Sri Lanka), (3) studies including adults 18 years of age and over, (4) studies that confirmed NAFLD with an ultrasound imaging and (5) studies published in the English language. In addition, we also included studies reporting NAFLD prevalence among participants with metabolic conditions, such as morbid obesity, diabetes mellitus, metabolic syndrome, and polycystic ovarian disease. The exclusion criteria were: (1) articles in a non-English language, (2) review articles and guidelines, and (3) clinical trials.

### Selection process

All the selected studies fulfilled the diagnostic criteria for NAFLD. This included fatty liver detected by ultrasound imaging and exclusion of causes of secondary fatty liver, including unsafe alcohol intake.

Two authors (MAN, MYW) independently screened the title and abstract of all the studies identified to select potentially eligible studies for the meta-analysis. Full texts of eligible articles were retrieved and studied in depth. The final decision on the inclusion of an article was in line with the agreed criteria, and a third author (DSE) resolved disagreements and disparities during the process.

The meta-analysis was conducted in two main steps: (i) pooling the prevalence of NAFLD and (ii) pooling the effect sizes of pre-defined associations of NAFLD (Fig. 1). In the first step (pooling the prevalence of NAFLD), descriptive and case-control studies reporting the NAFLD prevalence were selected. After that, the studies were divided into sub-analyses, where possible, by the setting (as “urban” or “rural”), by metabolic status (those with and without metabolic disease) and by the presence or absence of obesity. Missing data for one study^10^ was extracted from the relevant prevalence study on NAFLD in the same cohort.^11^ Exclusion criteria for the meta-analysis were: studies where NAFLD was diagnosed by methods other than ultrasound (liver biochemistry or histology), case-control studies, studies of NASH in NAFLD populations and studies which precluded the classification of participants into required categories or settings. In the second step (pooling the effect sizes of pre-defined associations of NAFLD), available data for associations of NAFLD were extracted from the selected studies.

### Data extraction

The following data were extracted from the studies: author name, year of publication, study designs, sample size, age group of participants (>18 years), study setting, diagnostic criteria used for diagnosing NAFLD, characteristics of the study population, and risk category of the participants. Data from selected studies were extracted to estimate pooled rates for the NAFLD prevalence and associations. Two authors performed data extraction independently (MAN, MYW), and any discrepancies were resolved by discussion with a third author (DSE).

Based on the available data, the selected studies were categorised as having a “general population” or “individuals having metabolic diseases”. The “general population” studies had chosen participants from the community with a presumed “average risk” of NAFLD. On the other hand, studies with “individuals having metabolic diseases” had participants with a metabolic derangement (diabetes mellitus/pre-diabetes mellitus, obesity, metabolic syndrome, cardiovascular disease, women with PCOD) with presumed “high risk” for NAFLD. The studies that were identified as “general population” were further divided into “rural” and “urban”.

Factors associated with NAFLD were extracted from the selected articles, after which, frequently reported factors were considered for pooling in the second step of the study. The selected factors were: male gender, general obesity, central obesity, diabetes mellitus, dysglycaemia, hypertension, dyslipidaemia and metabolic syndrome. Since studies reported different forms of glycaemic abnormalities, the term “dysglycaemia” was also used to combine diabetes mellitus, hyperglycaemia, and insulin resistance. However, the cut-off values for dyslipidaemia varied across the selected articles. Therefore, the participants, (i) who were already categorised as having hypertriglyceridemia/dyslipidaemia/high triglyceride (TG) levels or low high-density lipoproteins (HDL) on treatment, (ii) TG levels above 150 mg/dL or (iii) HDL below 40 mg/dL were included in the category of dyslipidaemia. As varying definitions have been used for general obesity, the participants who were directly categorised as obese in the study and participants with a BMI above the Asia-Pacific cut-off value (>25 kgm^−2^) were included in the general obese category. Similarly, participants directly categorised as having central obesity and participants with “high” waist circumference (WC > 90 cm in females and WC > 80 cm in males) were included in the category of central obesity.

### Quality assessment for individual studies

The quality of the studies was assessed using Joanna Briggs Institute Critical Appraisal Checklist for Prevalence Studies.^12^ This tool comprises nine questions that consider the following: sample frame appropriateness, recruitment appropriateness, sample size, descriptions of subjects and setting, coverage of data analysis, ascertainment and measurement of the condition, appropriateness of statistical analysis and the adequacy and management of the response rate. Each question had the option to answer ‘yes’, indicating higher quality; ‘no’, indicating poor quality; ‘unclear’ or ‘not applicable’. Two authors (SD, DSE) completed quality assessments for the studies considered eligible for inclusion. Any discrepancies in judgements regarding inclusion were resolved through discussion. Based on overall quality, studies with a number of positive responses (‘yes’) greater than six were included in the systematic review and meta-analysis. If a study had ≥3 ‘no’ or ‘unclear’ quality categories, it was excluded from the analysis. The appraisal outcomes were presented in a table (Table 1).

**Table 1:** Critical appraisal result using Joanna Briggs Institute (JBI) critical appraisal checklist for prevalence studies.

Ref. no	Study	Q1	Q2	Q3	Q4	Q5	Q6	Q7	Q8	Q9	# Yes (✓)	%Yes (✓)	Overall appraisal
^10^	Vendhan et al., 2014b	✓	✓	✓	✓	✓	✓	✓	✓	✓	9	100%	Include
^11^	Mohan et al., 2009	✓	✓	✓	✓	✓	✓	✓	✓	✓	9	100%	Include
^13^	Das et al., 2010	✓	✓	✓	✓	✓	✓	✓	x	✓	8	88.90%	Include
^14^	Pinidiyapathirage et al., 2011	✓	✓	✓	✓	✓	✓	✓	x	✓	8	88.90%	Include
^15^	Dassanayake et al., 2009	✓	✓	✓	✓	✓	✓	✓	x	✓	8	88.90%	Include
^16^	Alam et al., 2018	✓	✓	✓	✓	✓	✓	✓	✓	✓	9	100%	Include
^17^	Fahim et al., 2020	✓	✓	✓	✓	✓	✓	✓	✓	✓	9	100%	Include
^18^	Rahman et al., 2020	✓	✓	✓	✓	✓	✓	✓	✓	✓	9	100%	Include
^19^	Alam et al., 2013	✓	x	x	✓	✓	✓	✓	x	✓	6	66.70%	Include
^20^	Amarapurkar et al., 2007	✓	✓	✓	✓	✓	✓	✓	x	✓	8	88.90%	Include
^21^	Karla et al., 2013	✓	✓	✓	✓	✓	x	✓	x	✓	7	77.80%	Include
^22^	Vendhan et al., 2014 a	✓	✓	✓	✓	✓	✓	✓	x	✓	8	88.90%	Include
^23^	Praveenraj et al., 2015	✓	x	x	✓	✓	✓	✓	x	✓	6	66.70%	Include
^24^	Majumdar et al., 2016	✓	✓	✓	✓	✓	✓	✓	✓	✓	9	100%	Include
^25^	Anurag et al., 2015	✓	x	✓	✓	✓	✓	✓	x	✓	7	77.80%	Include
^26^	Ajmal et al., 2014	✓	x	x	✓	✓	✓	✓	x	✓	6	66.70%	Include
^27^	Uchil et al., 2009	✓	x	✓	✓	✓	✓	?	x	✓	6	66.70%	Include
^28^	Rajput et al., 2019	✓	x	x	✓	✓	✓	✓	x	✓	6	66.70%	Include
^29^	Kubihal et al., 2021	✓	x	✓	✓	✓	✓	✓	✓	✓	8	88.90%	Include
^30^	Harsha Varma et al., 2019	✓	x	x	✓	✓	✓	✓	x	✓	6	66.70%	Include
^31^	Atri et al., 2020	✓	x	x	✓	✓	✓	✓	✓	✓	7	77.80%	Include
^32^	Shrestha et al., 2019	✓	x	✓	✓	✓	✓	✓	x	✓	7	77.80%	Include
^33^	Paudel et al., 2019	✓	✓	✓	✓	✓	✓	✓	✓	✓	9	100%	Include
^34^	Iftikhar et al., 2015	✓	✓	✓	✓	✓	✓	✓	x	✓	8	88.90%	Include
^35^	Ghani et al., 2017	✓	x	✓	✓	✓	x	?	✓	✓	6	66.70%	Include
^36^	Shah et al., 2018	✓	x	x	✓	✓	✓	✓	x	✓	6	66.70%	Include
^37^	Hamid et al., 2019	✓	x	✓	✓	✓	✓	✓	x	✓	7	77.80%	Include
^38^	Abbas et al., 2013	✓	x	✓	✓	✓	✓	✓	x	✓	7	77.80%	Include
^39^	Taseer et al., 2009	✓	✓	x	✓	✓	✓	?	x	✓	6	66.70%	Include
	Bano et al., 2008	✓	x	x	✓	✓	✓	?	x	✓	5	55.60%	Exclude
^40^	Herath HMM et al., 2019	✓	x	x	✓	✓	✓	✓	x	✓	6	66.70%	Include
^41^	Herath RP et al., 2019	✓	✓	✓	✓	✓	✓	✓	✓		8	100%	Include
^42^	Perera et al., 2016	✓	✓	x	✓	✓	✓	✓	x	✓	7	77.80%	Include

Questions (Q) used in the checklist–Q1: Was the sample frame appropriate to address the target population? Q2: Were study participants sampled in an appropriate way? Q3: Was the sample size adequate? Q4: Were the study subjects and the setting described in detail? Q5: Was the data analysis conducted with sufficient coverage of the identified sample? Q6: Were valid methods used for the identification of the condition? Q7: Was the condition measured in a standard, reliable way for all participants? Q8: Was there appropriate statistical analysis? Q9: Was the response rate adequate, and if not, was the low response rate managed appropriately?

✓–“Yes”, x–“No”, ?–“unclear”.

### Statistical analysis and visualisation tools

In South Asia, the prevalence rates of NAFLD, diagnosed by ultrasound, ranged from 8.7% to 73.6%. The data showed differences in the study population (i.e., general and people with metabolic diseases), gender (male and female) and study settings (i.e., urban and rural). Therefore, the overall NAFLD prevalence (pooled estimates) across the studies was determined by performing a random-effects meta-analysis of proportions using the Der Simoniane Laird model. The number of NAFLD patients amongst the total number sampled in each study was considered for the analysis, and inverse variance weighting was used to pool the studies.

The Cochran Q test was used to assess the heterogeneity between the studies. Meta-analysis of the prevalence rates showed a high heterogeneity (I^2^ = 98.8%, Q = 1845.36, DF = 22, P < 0.001). The overall NAFLD prevalence was 40.4% (95% CI: 33.1–47.9%) when all the studies were considered together. There were 11 studies representing the “general population” (12,512 participants and 3374 NAFLD cases) and 12 studies from “individuals with metabolic disease” (2943 participants and 1367 NADLF cases), including three studies with overweight or obese NAFLD populations and one with a morbidly obese population. Subgroup analysis showed a difference in the prevalence rates between the general population and people with metabolic diseases and the difference was found to be statistically significant (Q = 15.8, DF = 1, P < 0.001). Therefore, a separate analysis was conducted for the general population and people with metabolic diseases (Supplementary Fig. S1).

Further, prevalence rates were evaluated for the differences in gender and study setting (i.e., rural versus urban) within general and urban populations. Subsequently, the reported associated factors (i.e., gender, general obesity, central obesity, diabetes mellitus, dysglycaemia, hypertension, dyslipidaemia, and metabolic syndrome) were evaluated to obtain pooled estimates for risk ratios using random-effects models. This was done by conducting a meta-analysis of binary variables where the number of NAFLD cases amongst people with and without the given risk factor was evaluated. Forest plots were developed to summarise the results of the meta-analysis, and the publication bias was assessed and visualised using a funnel plot. A P value of 0.05 was considered statistically significant. Data was analysed using R programming language 3.6.3 and a meta-analysis of proportions was carried out using the “meta” library. Outliers and influential cases were identified using “dmetar” library. Risk of bias plots and traffic light plots were obtained from “robvis” library.

### Role of the funding source

Not applicable.

## Results

### Selection of articles

A total of 158 articles were identified during the initial literature search. We included descriptive epidemiological studies with satisfactory methodological quality, reporting the prevalence of NAFLD with a valid diagnostic method.

Only 22 publications (two from Bangladesh, eleven from India, four from Sri Lanka, four from Pakistan and one from Nepal) were included in the subsequent meta-analysis after the quality assurance process (Fig. 1, Table 2). Studies included in the meta-analysis used only ultrasound/CT imaging to determine the presence of NAFLD. The meta-analysis included 15,455 study participants and 4741 NAFLD cases. Two studies had only female participants, while one had only male participants. There were 7280 males, including 2062 NAFLD cases and 7209 females, including 2035 NAFLD cases.

**Table 2:** Included studies on prevalence of non-alcoholic fatty liver disease in South Asia.

Country	Reference	Type	Population category	Mode of diagnosis
1) Bangladesh	Alam et al., 2018^16^	Cross sectional	Urban and rural community	Ultrasound
2) Bangladesh	Rahman et al., 2020^18^	Cross sectional	Rural community	Ultrasound
3) India	Amarapurkar et al., 2007^20^	Cross sectional	Urban community	Ultrasound
4) India	Das et al., 2010^13^	Prospective cohort study	Rural community	Ultrasound & CT
5) India	Vendhan et al., 2014a^22^	Retrospective cohort study	Hospital	Ultrasound
6) India	Majumdar et al., 2016^24^	Cross sectional	Rural community	Ultrasound
7) India	Anurag et al., 2015^25^	Cross sectional	Hospital	Ultrasound
8) India	Ajmal et al., 2014^26^	Cross sectional	Hospital	Ultrasound
9) India	Mohan et al., 2009^11^	Cross sectional	Urban community	Ultrasound
10) India	Vendhan et al., 2014b^10^	Cross sectional	Urban community	Ultrasound
11) India	Rajput & Ahlawat, 2019^28^	Cross sectional	Hospital	Ultrasound
12) India	Harsha Varma et al., 2019^30^	Cross sectional	Hospital	Ultrasound
13) India	Atri et al., 2020^31^	Cross sectional	Hospital	Ultrasound
14) Nepal	Paudel et al., 2019^33^	Cross sectional	Urban community	Ultrasound
15) Pakistan	Iftikhar et al., 2015^34^	Cross sectional	Hospital	Ultrasound
16) Pakistan	Hamid et al., 2019^37^	Cross sectional	Hospital	Ultrasound
17) Pakistan	Abbas et al., 2013^38^	Cross sectional	Urban community	Ultrasound
18) Pakistan	Taseer et al., 2009^39^	Cross sectional	Hospital	Ultrasound
19) Sri Lanka	Pinidiyapathirage et al., 2011^14^	Cross sectional	Rural community	Ultrasound
20) Sri Lanka	H. M. M. Herath et al., 2019^40^	Cross sectional	Hospital	Ultrasound
21) Sri Lanka	Dassanayake et al., 2009^15^	Prospective cohort study	Urban community	Ultrasound
22) Sri Lanka	Perera et al., 2016^42^	Cross sectional study	Hospital	Ultrasound

### Assessment of methodological quality

**Fig. 2:**
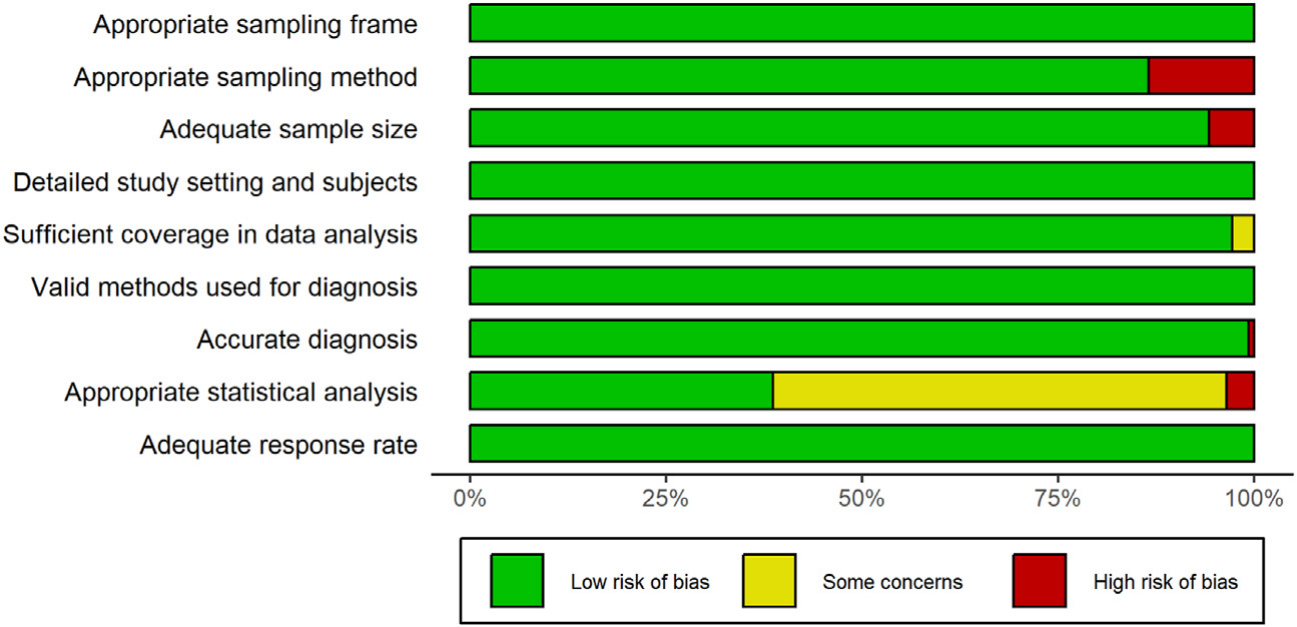
**Risk of bias graph: review of judgements of authors regarding each risk-of-bias item presented as percentages across included studies**.

**Fig. 3:**
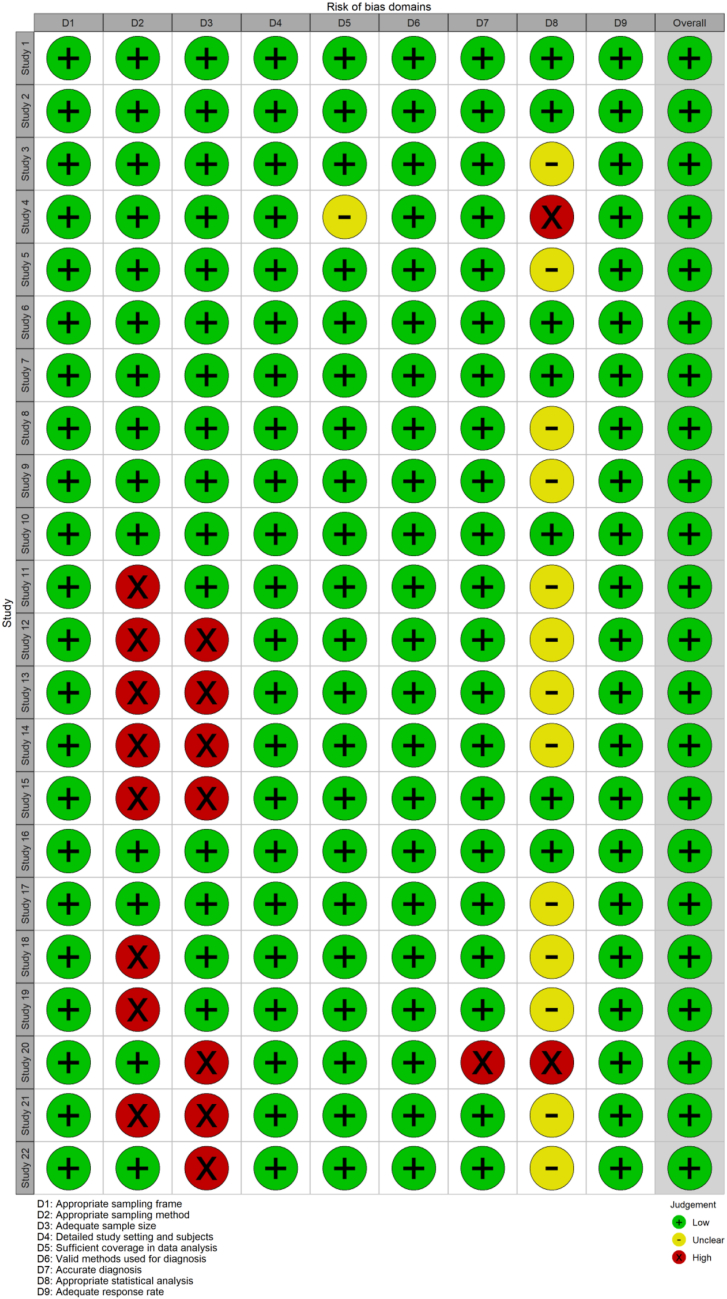
**Risk-of-bias summary: the judgements of authors regarding each risk-of-bias item for each included study**.

The quality assessment of the selected studies for the meta-analysis is presented in Figs. 2 and 3. The majority of the studies had a low risk of bias in each of the nine domains of the Joanna Briggs Institute Prevalence Critical Appraisal Tool, 2014.^12^

### Prevalence of NAFLD in the general population

**Fig. 4:**
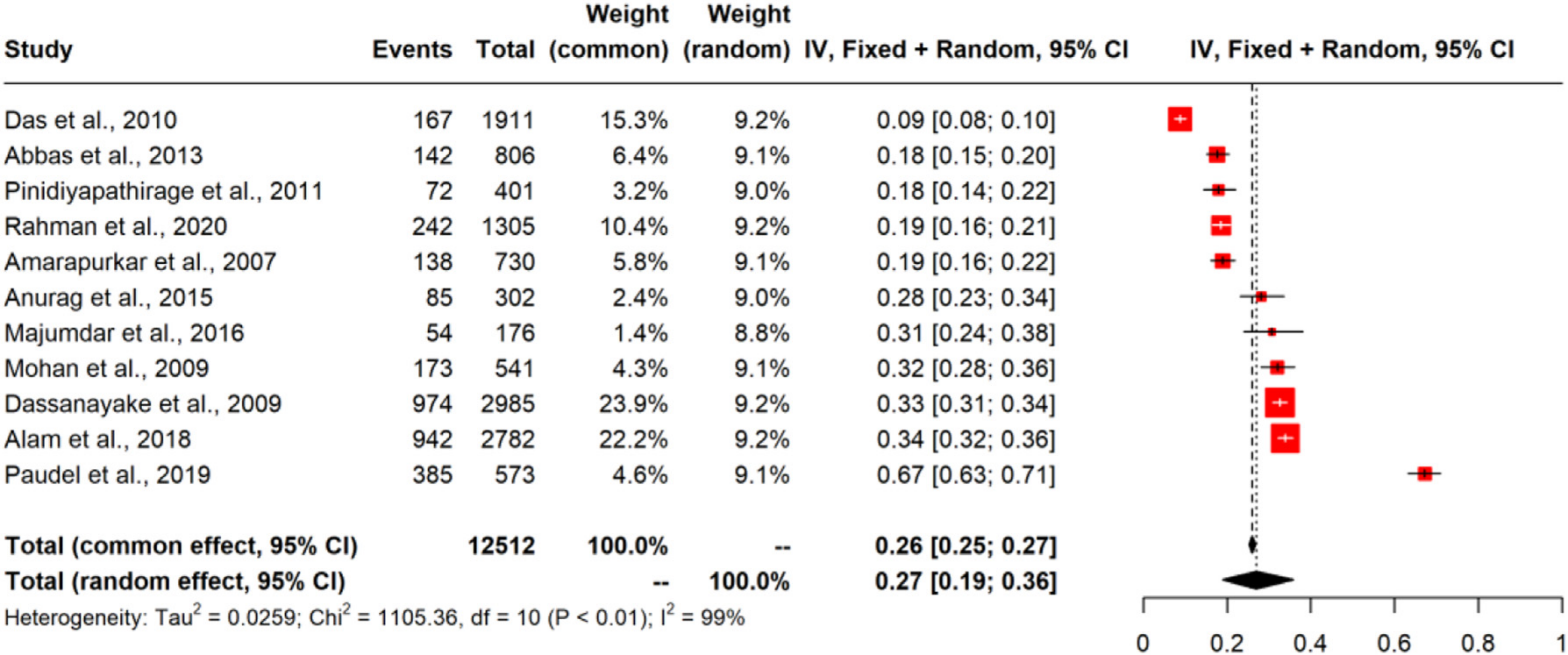
**Forest plot of non-alcoholic fatty liver disease (NAFLD) prevalence rates in the general population**.

Eleven studies reported NAFLD prevalence rates in the general population from Bangladesh, India, Nepal, Pakistan, and Sri Lanka. There were 12,512 individuals, including 3374 NAFLD cases. The pooled overall prevalence of NAFLD in the general population was estimated as 26.9% (95% CI: 18.9–35.8%) (Fig. 4). The prevalence rates showed high heterogeneity (k = 11; Q = 1105.36; DF = 10; P < 0.0001; I^2^ = 99.1%).

The general population included 5939 males and 6000 females. The overall NAFLD prevalence among males was 23.9% (95% CI: 17.6–31.0%), and among females was 22.7% (95% CI: 15.3–31.3%). The subgroup analysis did not show gender differences in the NAFLD prevalence rates (Q = 0.06, DF = 1, P = 0.806) (Supplementary Fig. S2). There were 4759 participants in rural settings and 7753 participants in urban settings. The NAFLD prevalence in the rural communities was 22.6% (95% CI: 13.6–33.1%), and it was 32.9% (95% CI: 22.8–43.8%) in urban communities without statistically significant differences in the prevalence rates (Q = 1.92, DF = 1, P = 0.166) (Supplementary Fig. S3).

### Prevalence of NAFLD in people with metabolic disease

**Fig. 5:**
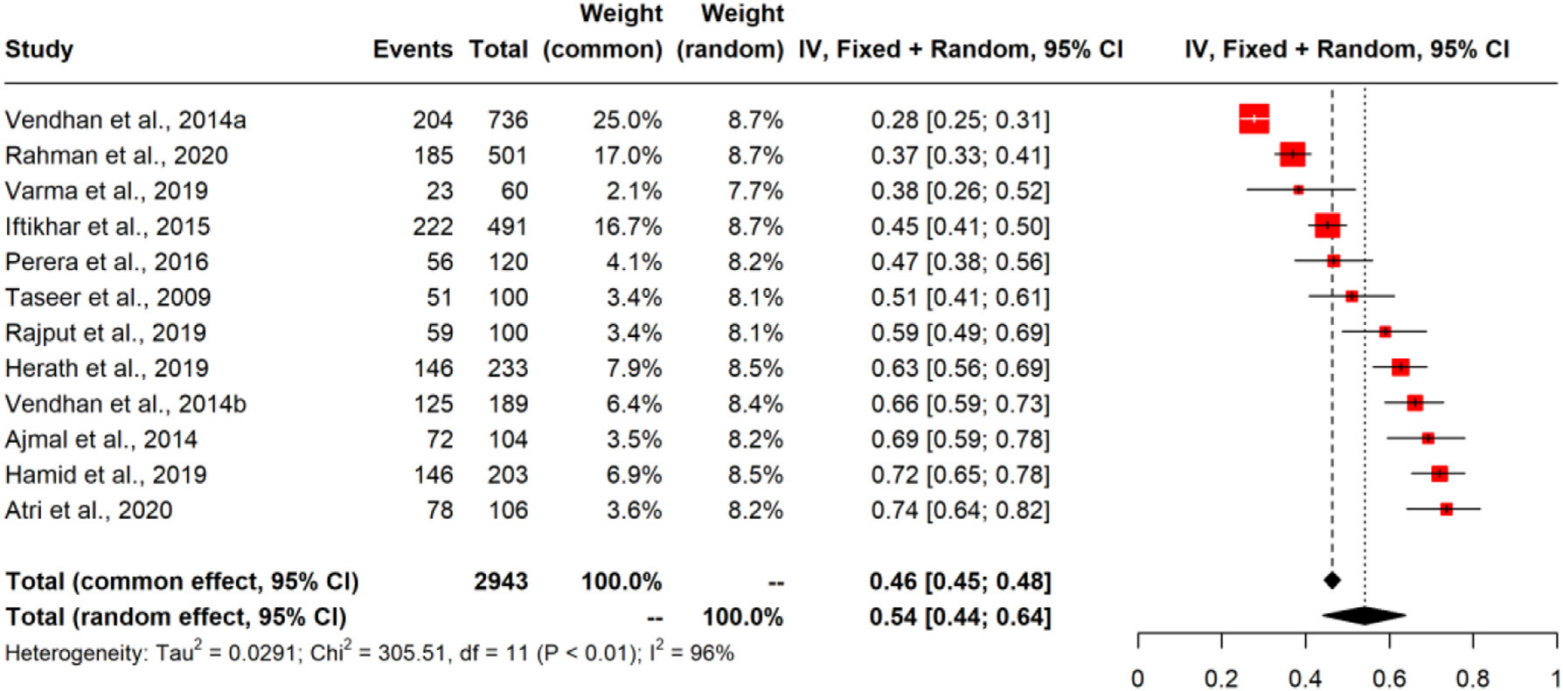
**Forest plot of non-alcoholic fatty liver disease (NAFLD) prevalence rates in the population with metabolic diseases**.

Twelve studies reported NAFLD prevalence rates in people with metabolic disorders from Bangladesh, India, Pakistan, and Sri Lanka. There were 2943 individuals, including 1367 NAFLD cases. The pooled overall prevalence of NAFLD in patients with metabolic diseases was 54.1% (95% CI: 44.1–63.9%) (Fig. 5). The prevalence rates showed high heterogeneity (k = 12; Q = 305.5; DF = 11; P < 0.0001; I^2^ = 96.4%).

From these studies, data were available for 1209 males and 1341 females. One study had only males,^34^ few studies had only females,^30^^,^^31^ whilst few studies did not provide data on gender.^26^^,^^28^ The subgroup analysis showed the overall NAFLD prevalence was 48.6% (95% CI: 38.2–59.1%) in males and 53.1% (95% CI: 37.2–68.6%) in females, without statistically significant difference in the prevalence rates with respect to gender (Q = 0.21, DF = 1, P = 0.647) (Supplementary Fig. S4).

### Non-obese NAFLD

**Fig. 6:**
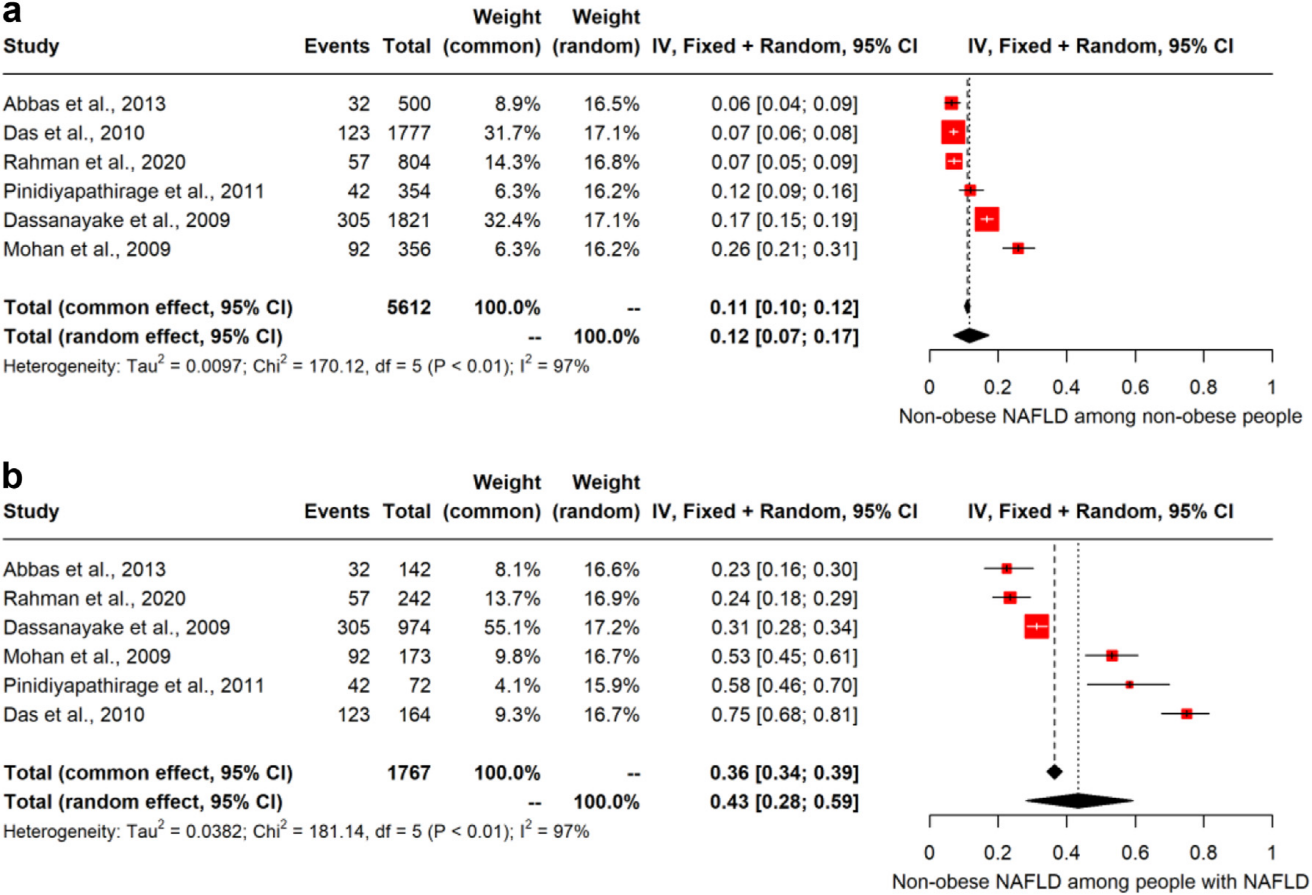
**Pooled prevalence of non-obese NAFLD [non-alcoholic fatty liver disease] in (a) non-obese people and (b) people with NAFLD**.

Six studies provided data on non-obese NAFLD, which included 5612 non-obese participants and 651 non-obese NAFLD cases. The pooled overall estimate for prevalence of NAFLD in the non-obese population was 11.7% (95% CI: 7.0–17.3%) (k = 6; Q = 170.1; DF = 5; P < 0.0001; I^2^ = 97.1%, Fig. 6a). These six studies included a total 1767 NAFLD cases. The pooled estimate for the prevalence of non-obese in the NAFLD population was 43.4% (95% CI: 28.1–59.4%) (k = 6; Q = 181.1; P < 0.0001; I^2^ = 97.2%, Fig. 6b).

### Associations for NAFLD

Meta-analysis of binary variables showed that the presence of NAFLD in the South Asian population was associated with diabetes mellitus, hypertension, dyslipidaemia, general obesity, central obesity, and metabolic syndrome. Gender was not associated with NAFLD. The estimated relative risk (RR) for each associated factor from random effect models is shown in Table 3.

**Table 3:** Pooled estimates of relative risk of associated factors with non-alcoholic fatty liver disease in South Asia.

Variable (Condition)	Number of studies considered	No. of individuals with NAFLD + condition/Total individuals with condition	No. of individuals with NAFLD but without condition/Total individuals without condition	RR	95% CI	P value
Diabetes mellitus	11	509/938	1420/5826	2.03	1.56–2.63	<0.0001
Dysglycemia	14	1061/2478	2078/9594	1.61	0.99–2.61	0.0532
Hypertension	11	633/1369	1300/4807	1.37	1.03–1.84	0.0316
Dyslipidemia	11	1136/3125	889/3311	1.68	1.51–1.88	<0.0001
General obesity	14	1868/3610	1634/9367	2.56	1.86–3.51	<0.0001
Central obesity	7	804/1848	434/3833	2.51	1.69–3.72	0.0001
Metabolic syndrome	6	572/1226	330/2316	2.86	1.79–4.57	<0.0001
Male gender	18	899/7066	2020/7555^a^	1.09	0.89–1.21	0.618

RR—relative risk.

a Female gender.

## Discussion

This meta-analysis involved 22 studies, with 15,455 participants and 4741 NAFLD cases from five South Asian countries (Bangladesh, India, Nepal, Pakistan, and Sri Lanka). We estimated that the pooled prevalence of NAFLD among adults in the general population was 26.9%. Our estimated pooled prevalence of NAFLD in South Asia is comparable to the estimated global prevalence of 25.4%.^1^

Estimating the prevalence of NAFLD in the general population (with average risk) is challenging as the disease is mainly asymptomatic. However, the current study determined that the overall prevalence of NAFLD among the general population, excluding those with metabolic diseases, was 26.9%. This finding highlights the high proportion of apparently metabolically normal people in the community with NAFLD. Furthermore, there was no difference between prevalence estimates for NAFLD in the current study between males and females as well as residents of urban and rural communities.

In the current meta-analysis, the pooled prevalence of NAFLD among individuals with metabolic diseases with high risk was 54.1%, significantly higher than the prevalence in the general population. The pooled prevalence of NAFLD among individuals with metabolic disorders is comparable with the estimated global prevalence of NAFLD among patients with Type 2 diabetes mellitus, 55.5%.^43^ Furthermore, pooling estimates for the relative risk values of NAFLD showed a significant association with general and central obesity, diabetes mellitus, hypertension, dyslipidaemia, and metabolic syndrome, confirming the strong association between metabolic abnormalities and NAFLD in this South Asian population.

A major proportion of South Asian NAFLD patients are non-obese. This paradox^44^^,^^45^ is related to ethnic disparities and visceral fat distribution.^46^^,^^47^ In the present study, the pooled estimate for the prevalence of NAFLD in the non-obese population was 11.7%, comparable with prevalence rates reported in high-income countries (HICs).^48^, ^49^, ^50^ However, the prevalence of non-obese NAFLD among the NAFLD population in the present study was 43.4%, which is far higher than rates reported in HICs.^13^^,^^14^, ^51^ However, the prevalence of non-obese NAFLD in the HICs and low-income and middle-income countries may not be directly comparable, at least partly due to the different BMI cut-offs,^52^ which could have contributed to the dissimilarity observed during the current study. South Asian patients with normal BMI (<23 kg/m^2^) may still have central obesity, evidenced by increased WC, reflecting an increase in visceral fat. Studies have demonstrated that visceral fat, originally considered a passive depot for energy storage, is an active endocrine tissue that releases many mediators that regulate metabolism, inflammation, and immunity that is also involved in the pathogenesis of NAFLD. The increase in visceral fat is closely related to the presence of fatty liver, liver inflammation and fibrosis.^53^^,^^54^ Therefore, a high index of suspicion is necessary not to miss non-obese NAFLD in clinical practice. It is also noteworthy that non-obese NAFLD is an independent risk factor for coronary artery disease among the South Asian populations.^10^, ^15^^,^^44^

Before the present study, only a limited number of studies evaluated the prevalence of NAFLD in South Asia. A review article on NAFLD in South Asia in 2016 estimated the epidemiology and determinants of NAFLD in different South Asian countries, including India, Sri Lanka, Bangladesh, Pakistan and Nepal.^9^ The prevalence of NAFLD in South Asia was reported to vary from 9% to 45%. The lowest prevalence of NAFLD (8.7–18%) was observed in physically active, poor, lean individuals from rural regions.^44^^,^^45^ Obesity, acanthosis nigricans, fasting hyperglycaemia, transaminitis, male sex, high BMI, high WC, and hypertension were factors found to be associated with NAFLD.^45^^,^^46^ A recent Asian Pacific Association for the study of the liver (APASL) guideline recommends the adoption of the term metabolic dysfunction-associated fatty liver disease (MAFLD) to replace NAFLD.^55^ A much higher (nearly double) pooled prevalence of NAFLD among individuals with MetD (with high risk) compared the general population, excluding those with MetD (with average risk) is reflective and supportive of the proposed term MAFLD. Unfortunately, we could not extract the necessary data to diagnose MAFLD in the populations described in the studies included in the systematic review and meta-analysis. Therefore, the analysis was limited to NAFLD in the present paper.

The most recent review article on NAFLD in South Asians, published in 2017, was based on a comprehensive search of available articles on NAFLD in South Asian countries from 1980 to 2016.^8^ Estimations of NAFLD across South Asian countries were demonstrated via mapping and tables. A salient finding was that NAFLD was prevalent in cities and rural areas. Among the factors with statistically significant associations were age, obesity, insulin resistance, and metabolic syndrome. In addition, a higher prevalence of all components of metabolic syndrome was seen among NAFLD cases. The findings reported in these reviews are similar to the result of the present study.

At the time of the final analysis of our study, a systemic review and meta-analysis of the prevalence of NAFLD in India was published by Lee and colleagues.^54^ This was the first meta-analysis of all published studies on NAFLD in India. The study participants included children and adults, with an estimated sample of 26,484 from 50 published studies (children, n = 2093; adults, n = 23,581). While this had a similar methodology to our research, it assessed the prevalence of NAFLD within the population sub-categorised into average-risk and high-risk groups. The high-risk group consisted of obesity, overweight, pre-diabetes, diabetes mellitus, coronary artery disease, metabolic syndrome, obstructive sleep apnoea, polycystic ovarian syndrome and elevated liver enzymes. Notably, the high-risk group had a higher prevalence of NAFLD at 52.8%, comparable to the estimated 54.1% in the present study for those with NAFLD in the presence of associated metabolic disease.^56^ Conversely, those in the average-risk group were noted to have a lower prevalence of NAFLD at 28.2% compared to our estimate of 26.9%.^56^ Despite the significant heterogeneity among studies and sampling bias, the above findings of the study by Lee and colleagues^54^ reflect similarly in our study, which found a statistically significant association between NAFLD and metabolic syndrome, general obesity, central obesity, diabetes, dysglycaemia, dyslipidaemia and hypertension. Notably, the high prevalence of NAFLD in India and that of the South Asian region, as noted in our study, further highlights the growing public health concern for the region.

Another recent study by Kam and colleagues reported clinical profiles of Asians with NAFLD.^57^ This study reported relevant data, especially BMI and alanine transaminase (ALT), among nearly 2.25 million Asians with NAFLD. About one-third of Asians with NAFLD were non-obese, and the majority did not have markedly elevated ALT. Therefore, Kam and colleagues^57^ concluded that abnormal ALT or BMI is not recommended as a criterion for NAFLD screening in this population. In the present study, we report an even higher percentage of non-obese NAFLD among South Asians (43.4%). This may be mainly due to higher proportions of South Asia individuals with NAFLD having central obesity (increased WC) rather than general obesity (raised BMI).

Our study has several strengths and limitations. The present study is the only meta-analysis on the prevalence of NAFLD in the South Asian region and the meta-analysis comprehensively assessed the available data over 17 years (2004–2021). We have performed sub-analyses for the general population, rural and urban settings, non-obese populations, and those with metabolic abnormalities. For uniformity, we selected only studies that diagnosed NAFLD based on abdominal ultrasound for the meta-analysis. We conducted an extensive quality assessment of the studies included in the analysis. Only epidemiological studies with satisfactory methodological quality were included to minimise the heterogenicity and risk of bias.

A major limitation of our study is the lack of data from three South Asian countries (Afghanistan, Bhutan, and Maldives), which limits the generalisability of our findings beyond the countries included in the analysis. We avoided including further data from abstracted publications to expand the results across the region due to the potential lack of quality of those data. Also, the information on alcohol consumption was obtained by direct questioning of the participants in the studies included in the analysis. This may have led to under-reporting of alcohol use patterns with consequent overestimation of the prevalence of NAFLD. Furthermore, we could not evaluate age as a risk factor for NAFLD due to lack of individual data in most studies. All the studies considered for the meta-analysis did not include all the associated factors assessed for NAFLD. Therefore, we could only conduct univariable meta-analyses of associated factors as there were not sufficient data for a meta-regression analysis. Hence, the analysis of associated factors should be interpreted as exploratory analysis.

Furthermore, the current study was limited to available data among adults, excluding the paediatric and adolescent populations. According to the reported pooled prevalence of NAFLD in the paediatric and teenage population,^58^ it can be predicted that this population would contribute to the rising burden of NAFLD in the South Asian region. We could not assess the burden of significant liver fibrosis, which determines the outcomes in patients with NAFLD, as data was not reported in most studies. The high heterogeneity of the included studies was another limitation of the present study. Despite this limitation, the result is likely to represent the NAFLD burden in the South Asian region.

Elastographic techniques are widely adopted and recommended by guidelines for assessing NAFLD. Transient elastography (TE) permits the simultaneous measure of steatosis by controlled attenuation parameter (CAP) and liver fibrosis by liver stiffness measurement. A few studies on NAFLD using TE were not included in the systematic review, as the researchers believed insufficient studies used TE and CAP specifically conducted in the South Asian population.^59^^,^^60^

The prevalence of NAFLD among adults in the South Asian region seems comparable to the global average and a considerable proportion of adults were non-obese. NAFLD prevalence was notably higher among individuals with metabolic abnormalities. Diabetes mellitus, hypertension, dyslipidaemia, metabolic syndrome, and obesity (general and central) were identified as associated factors of NAFLD in the South Asian region. Well-designed community-based studies are needed to assess NAFLD prevalence across the South Asian region to plan resource allocation to mitigate the disease burden. Targeted health strategies should be implemented across the South-Asian region to address this growing public health problem.

## Contributors

MAN and DSE conceptualised the study. MAN, MYW, SD, DSE and HJdeS formulated the methodology. MAN, MYW, SD and STDeS extracted the data. DSE and SD analysed the data. MAN, SD and DSE drafted the manuscript. STDeS and HJdeS critically analysed and revised the manuscript. All authors read and approved the final manuscript.

## Data sharing statement

Data supporting this meta-analysis can be obtained through corresponding author on special request.

## Declaration of interests

None.
