# Supplementary material for: Prevalence and associated factors for non-alcoholic fatty liver disease among adults in the South Asian Region: a meta-analysis
Source: Lancet Reg Health Southeast Asia. 2023 May 24;15:100220. doi: 10.1016/j.lansea.2023.100220 (PMC10442973; doi:10.1016/j.lansea.2023.100220)
Supplement: Supplementary Figs. S1–S4 and Supplementary Table S1 [file mmc1.docx]

**Supplementary material**

**Supplementary Table 1: Characteristics of the included studies on prevalence of non-alcoholic fatty liver disease (NAFLD) in South Asia**

| **Country (region)** | **Reference** | **Predominant sex among NAFLD No (%)** | **Prevalence by sex**  **No (%)** | **Mean age**  **(Years ± SD)** | **BMI in kg/m2**  **(Mean** ± **SD)** | **Obesity**  **(%)** | **Dysglycemia (%)** | **MetS**  **(%)** | **Associations/Risk factors** |
| --- | --- | --- | --- | --- | --- | --- | --- | --- | --- |
| 1. Bangladesh | Alam et al., 2018 [34] | M 573(60.8) | M 573(33.8)  F 369(33.9) | 39.73 ± 11.71 | 26.61 ± 4.07 | NA | 16.3 | NA | Increasing age  Diabetes Mellitus  High BMI  Married status |
| 1. Bangladesh | Rahman et al., 2020 [36] | F 115(61.6) | M 93(23.4)  F 115(16.4) | 46.1 ± 12.3 | 27.93 ± 4.04 | 76.4 | 33.9 | 73.1 | Age >40 years  Male gender  MetS  Diabetes mellitus  Abdominal obesity  Hypertension  Dyslipidemia  Obesity |
| 1. India | Amarapurkar et al., 2007 [38] | M 84(60.9) | M 84 (24.6)  F 53(13.6) | 39.08 ± 12.23 | 26.6 ± 5.1 | 52 | 22 | NA | Age > 40 years  Male sex  Increased WC  BMI >25  AST, ALT more than 40 Iu/ML  High FBS |
| 1. India | Das et al., 2010 [23] | M 88 (53.6) | M 88(8.6)  F 76(8.5) | 39.0 ± 12.7 | 23.0 ± 4.2 | 25 | 26 | NA | BMI >25  Abdominal obesity  Dysglycemia  Higher income |
| 1. India | Vendhan et al., 2014a [40] | M 118(57.8) | M 118(30.7)  F 86(24.4) | 23 ± 11 | 20.2 ± 4.7 | 13.7 | NA | NA | Retinopathy and nephropathy |
| 1. India | Majumdar et al., 2016 [42] | F 43(79.6) | M 11(33.3)  F 43(30.1) | 54.4 ± 10.6 | NA | NA | NA | 9.3 | Hypertension  Increased WC |
| 1. India | Anurag et al., 2015 [43] | F 48(56.5) | M 37(27.4)  F 48(28.7) | 50.75 ± 14.06 | 25.38 ± 6.58 | NA | 48.2 | 36.5 | Increased BMI  Metabolic syndrome,  Increased FBS  Increased serum triglycerides |
| 1. India | Ajmal et al., 2014 [44] | NA | NA | 53.8 ± 11.7 | 23.7 ± 3.8 | 32.7 | 13.9 | 52.8 | High BMI  High serum triglyceride level  Increased WC  Hypertension |
| 1. India | Mohan et al., 2009 [11] | M 91(52.6) | M 91(35.1)  F 82(29.1) | NA | 25.2 ± 4.0 | 72.3 | 76.3 | 54.3 | Diabetes Mellitus  MetS |
| 1. India | Vendhan., et al., 2014b [10] | M 26(54.2) | NA | 46 ± 14 | 20.9 ± 1.7 | NA | NA | NA | Diabetes Mellitus  Hypercholesterolemia |
| 1. India | Rajput & Ahlawat, 2019 [46] | NA | NA | 43.42 ± 11.09 | 26.21 ± 2.57 | NA | All | 31.6 | GGT  High WC |
| 1. India | Harsha Varma et al., 2019 [48] | All Female | All Female | 23.91 ± 6.54 | 30.58 ± 5.82 | 87 | NA | NA | HOMA-IR  Hyper-androgenemia |
| 1. India | Atri et al., 2020 [49] | All Female | All Female | 40.9 ± 11 | 42.3 ±6.1 | All morbidly obese | NA | NA | High WC  High BMI  High Waist-Height Ratio |
| 1. Nepal | Paudel et al., 2019 [51] | M 209(54.2) | NA | NA | NA | NA | 45.7 | 57.6 |  |
| 1. Pakistan | Iftikhar et al., 2015 [52] | All Male | All Male | 41.3 ± 6.43 | 27.3 ± 0.67 | NA | NA | All |  |
| 1. Pakistan | Hamid et al., 2019 [55] | Equal | M 73(70.2)  F 73(73.7) | 52.44 ± 9.63 | NA | 85.6 | All | NA | Dyslipidemia  Higher LDL  High HbA1c  High DBP |
| 1. Pakistan | Abbas et al., 2013 [56] | M 81(57) | M 81(16.5)  F 61(19.4) | 43.3±12.1 | 28.6 ± 6.0 | 77.5 | 9.8 | NA | High BMI |
| 1. Pakistan | Taseer et al., 2009 [57] | M 32(62.75) | M 19(38.8)  F 32(62.7) | NA | NA | NA | All | NA |  |
| 1. Sri Lanka | Pinidiyapathirage et al., 2011 [24] | M 45(62.5) | M 45(23.8)  F 27(12.7) | 50.5 ± 7.9 | NA | 41.7 | 41.7 | NA | Male sex,  High BMI  High WC  High DBP  High FBS |
| 1. Sri Lanka | Herath et al., 2019 [58] | F 80(55) | M 67(60.1)  F 80(65) | 56.7 ± 8.9 | 25.5 ± 3.4 | 65 | All | NA | Use of Pioglitazone  Higher BMI  Increased WC |
| 1. Sri Lanka | Dassanayake et al., 2009 [27] | F 605(62.1) | M 369(27.2)  F 605(37.2) | 52.8 ± 7.3 | 27.1 ± 3.9 | 59.9 | 50.3 | NA | Obesity  Acanthosis nigricans  Insulin resistance  Elevated DBP  High FBS  High serum triglycerides  ALT ≥ twice upper limit |
| 1. Sri Lanka | Perera et al., 2016 [60] | M 30(53.6) | M 30(40)  F 26(57.8) | 62.9 ± 10.8 | 24.59 ± 3.5 12 | 21.5 | 67.9 | NA |  |

NAFLD, Non-Alcoholic Fatty Liver Disease; MetS, Metabolic Syndrome; BMI, Body Mass Index; HOMA-IR, Homeostatic Model Assessment-Insulin Resistance; GDM, Gestational Diabetes Mellitus; WC, Waist Circumference; LDL, Low Density Lipoprotein, HDL, High Density Lipoprotein, DM, Diabetes Mellitus; DBP, Diastolic Blood Pressure, FBS, Fasting Blood Sugar; ALT, Alanine Transaminase; GGT, Gamma Glutamyl Transferase

Reports Lean NAFLD

**
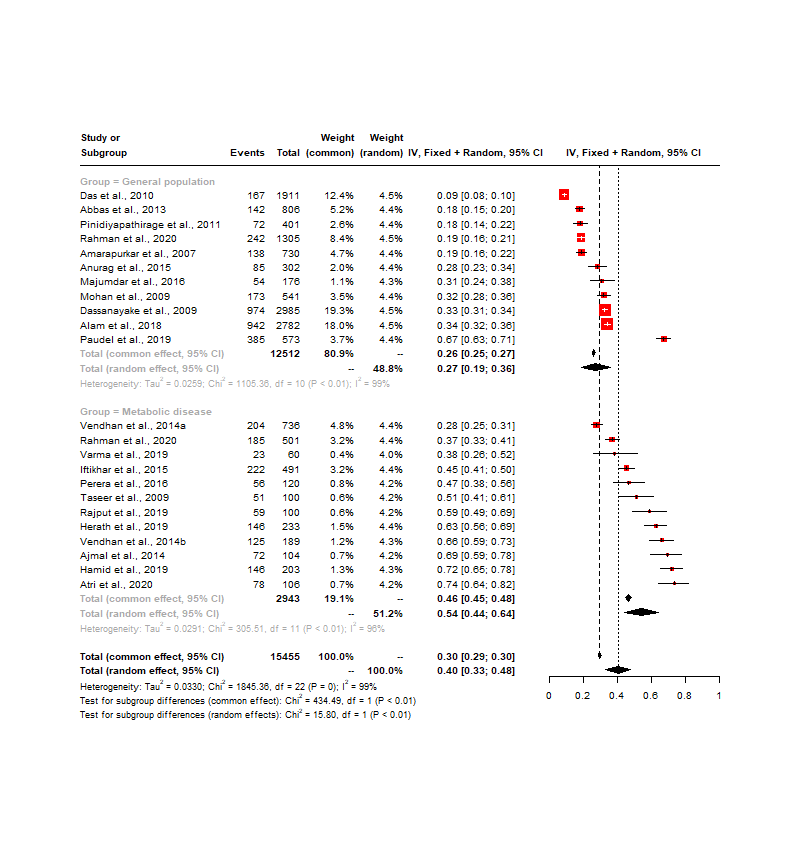
**

**Supplementary Figure 1: Subgroup analysis of general population and population with metabolic diseases. The population with metabolic diseases showed significantly higher prevalence rate compared to the general population.**

**
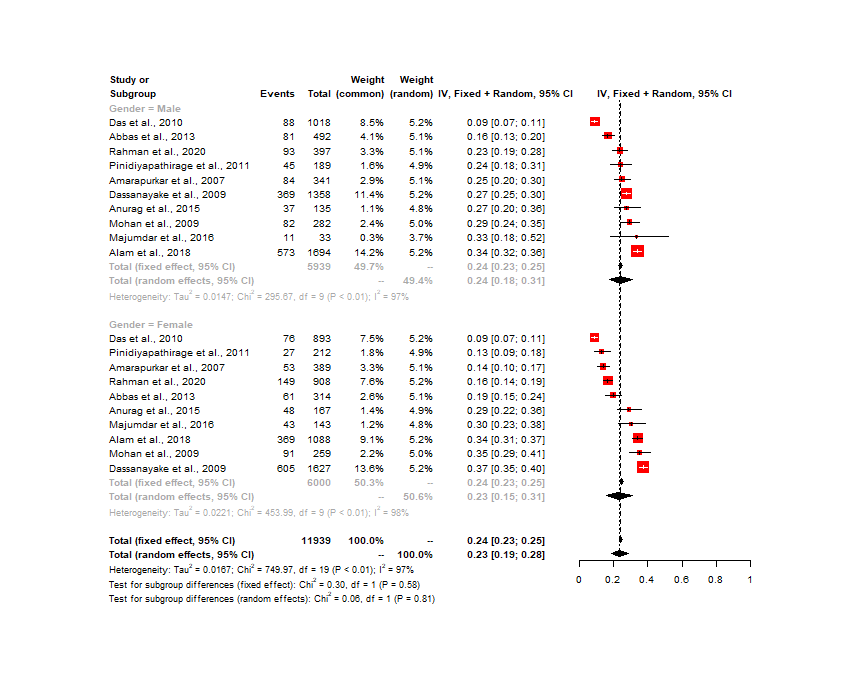
**

**Supplementary Figure 2: Subgroup analysis of males versus females in general population.**

**
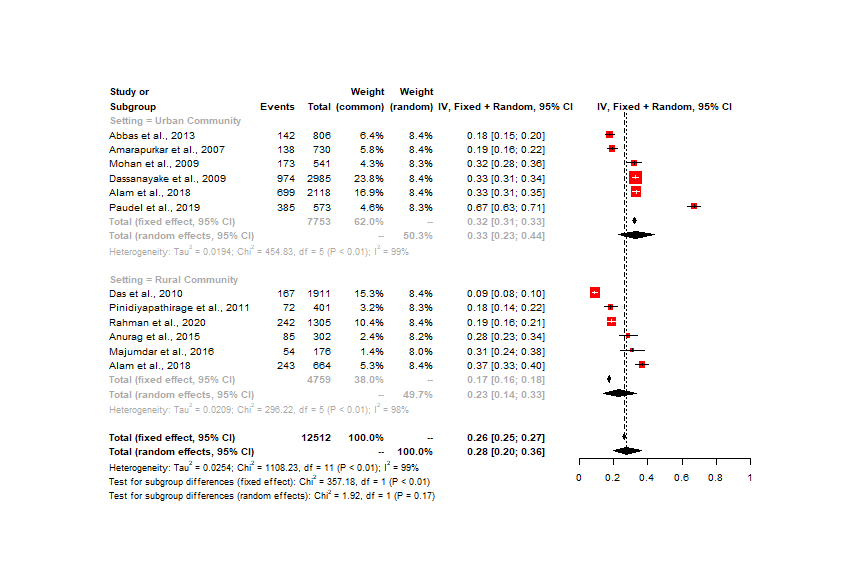
**

**Supplementary Figure 3: Subgroup analysis of urban versus rural settings in the general population.**

**
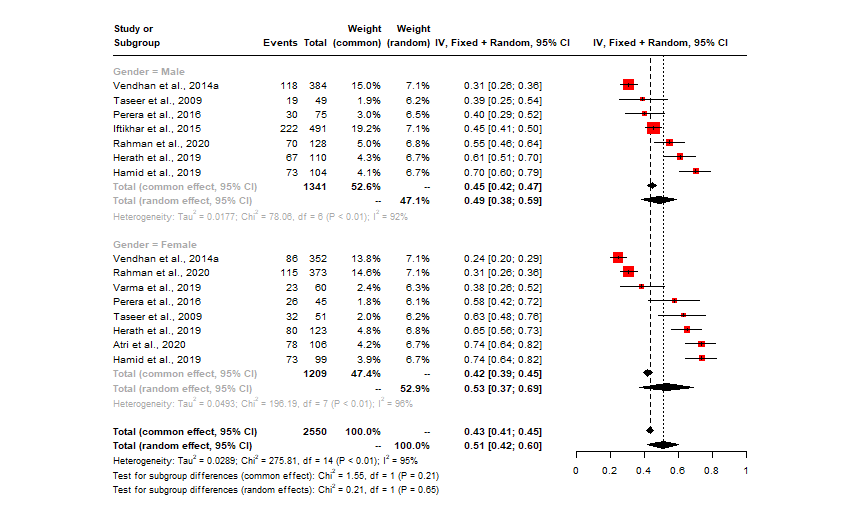
**

**Supplementary Figure 4: Subgroup analysis of males versus females in the population with metabolic diseases.**
